# Effects of n-3 fatty acids and exercise on oxidative stress parameters in type 2 diabetic: a randomized clinical trial

**DOI:** 10.1186/s12970-018-0222-2

**Published:** 2018-04-23

**Authors:** Ana Paula Trussardi Fayh, Katiuce Borges, Giovani Santos Cunha, Mauricio Krause, Ricardo Rocha, Paulo Ivo Homem de Bittencourt, José Cláudio Fonseca Moreira, Rogério Friedman, Juliane da Silva Rossato, Jõao Roberto Fernandes, Alvaro Reischak-Oliveira

**Affiliations:** 1 Departament of Nutrition, Health Sciences Center, Federal University of State of Rio Grande do Norte, Avenida Senador Salgado Filho, n° 3000, Natal, RN 59078-970 Brazil; 2 Laboratório de Pesquisa do Exercício, Escola de Educação Física, Universidade Federal do Rio Grande do Sul, Porto Alegre, RS 90690-200 Brazil; 3 Laboratory of Inflammation, Metabolism and Exercise Research (LAPIMEX) and Laboratory of Cellular Physiology, Departamento de Fisiologia, Instituto de Ciências Básicas da Saúde, Universidade Federal do Rio Grande do Sul, Porto Alegre, RS 90050-170 Brazil; 4 Departamento de Bioquímica, Instituto de Ciências Básicas da Saúde, Universidade Federal do Rio Grande do Sul, Porto Alegre, RS 90035-000 Brazil; 5 Endocrine Unit, Hospital de Clínicas de Porto Alegre, Universidade Federal do Rio Grande do Sul, Porto Alegre, RS 90035-000 Brazil

**Keywords:** Omega-3, Type 2 diabetes, Acute exercise, Oxidative stress, Inflammation

## Abstract

**Background:**

The relationship between diabetes and oxidative stress has been previously reported. Exercise represents a useful non-pharmacological strategy for the treatment in type 2 diabetic (T2DM) patients, but high intensity exercise can induce a transient inflammatory state and increase oxidative stress. Nutritional strategies that may contribute to the reduction of oxidative stress induced by acute exercise are necessary. The aim of this study was to examine if n-3 PUFA supplementation intervention can attenuate the inflammatory response and oxidative stress associated with high intensity exercise in this population. As a primary outcome, lipoperoxidation measurements (TBARS and F2-isoprostanes) were selected.

**Methods:**

Thirty T2DM patients, without chronic complications, were randomly allocated into two groups: placebo (gelatin capsules) or n-3 PUFA (capsules containing 180 mg of eicosapentaenoic acid and 120 mg of docosahexaenoic acid). Blood samples were collected fasting before and after 8 weeks supplementation. In the beginning and at the end of protocol, an acute exercise was performed (treadmill), and new blood samples were collected before and immediately after the exercise for measurements of oxidative stress and high-sensitivity C-reactive protein (hs-CRP).

**Results:**

After the supplementation period, a decrease in triglycerides levels was observed only in n-3 PUFA supplementation group (mean difference and 95% CI of 0.002 (0.000–0.004), *p* = 0.005). Supplementation also significantly reduced TRAP levels after exercise (mean difference and 95% CI to 9641 (− 20,068–39,351) for − 33,884 (− 56,976 - -10,793), *p* = 0.004, Cohen’s *d* effect size = 1.12), but no significant difference was observed in n-3 PUFA supplementation group in lipoperoxidation parameters as TBARS (mean difference and 95% CI to − 3.8 (− 10–2.4) for − 2.9 (− 1.6–7.4) or F2-isoprostanes (mean difference and 95% CI -0.05 (− 0.19–0.10) for − 0.02 (− 0.19–0.16), *p* > 0.05 for both.

**Conclusion:**

PUFA n-3 supplementation reduced triglycerides as well as TRAP levels after exercise, without a significant effect on inflammatory and oxidative stress markers.

This study is registered at ClinicalTrials.gov with the registration number of NCT03182712.

## Background

Hyperglycemia is the hallmark metabolic abnormality associated with the complications of type 2 diabetes mellitus (T2DM). High rates of glucose utilization are associated with incremental free radical formation and protein modification (by glucose auto-oxidation, protein glycation, and polyol pathway activation) [1]*.* The redox imbalance resulting from the increase of reactive oxygen and nitrogen species, and the concomitant lowering of antioxidant defenses have been associated to micro- and macrovascular diabetic complications [2, 3]. Nevertheless, the underlying mechanisms related to this imbalance are not entirely clear.

The relationship between hyperglycemia and increased lipid peroxidation has been previously reported [2, 4, 5]. Elevated levels of thiobarbituric acid reactive substances (TBARS) have been found in the blood of both type 1 [6] and type 2 [4] diabetic patients. The gold standard lipid peroxidation marker, F2-isoprostane, a toxic product generated by non-enzymatic, free radical-catalyzed peroxidation of arachidonic acid, has also been shown to be elevated in plasma and urine of T2DM [6, 7]. Disrupted redox signalling or elevated oxidative stress (from prolonged periods of hyperglycemia and/or elevated pro-inflammatory cytokines) is thought to underlie the vascular dysfunction observed in individuals with glucose intolerance and diabetes [3]. It has also been shown that individuals with T2DM have more pronounced systemic inflammation and oxidative stress than those with normal glucose tolerance [8]. In addition, inflammatory markers, such as high-sensitivity C-reactive protein (hs-CRP), are associated with higher risk for cardiovascular diseases and are also important markers for the control and prevention of diabetes complications [9].

Regular physical exercise and diet represents a useful non-pharmacological strategy for the prevention and treatment of T2DM [3]. The beneficial effects of chronic exercise are associated with improved mitochondrial viability and antioxidant defenses, reducing oxidative stress and provides acute and chronic benefits [8, 10, 11]**,** however, high intensity exercise can induce a transient inflammatory state and increase oxidative stress [10, 11]. Thus, nutritional strategies that may contribute to the reduction of oxidative stress induced by acute exercise may represent additional advantages to T2DM people.

Among many dietary components and supplements, studies have demonstrated beneficial effects of Polyunsaturated Fatty Acids Omega-3 (n-3 PUFA) on different conditions, including diabetes [12–14]. These effects include alterations in lipoprotein metabolism, platelet and endothelial function, and eicosanoid production. N-3 PUFA play an important role on cell membranes, are considered essential fatty acids for human beings, and should be provided by food intake [3]. Although fatty acid unsaturation favors free radical activity (increasing lipoperoxidation), the available literature is controversial when it relates to oxidative stress following n-3 PUFA supplementation [4, 15, 16].

Therefore, the aim of this study was to examine if n-3 PUFA supplementation intervention can attenuate the inflammatory response/oxidative stress associated with high intensity exercise in this population. As a primary outcome, lipoperoxidation measurements (TBARS and F2-isoprostanes) were selected. The antioxidant variables (superoxide dismutase, TRAP and uric acid) were considered secondary outcomes, and complement the oxidative stress responses. Additionally, biochemical changes in the lipid and glycemic profile before and after n-3 supplementation were investigated. We hypothesized that n-3 PUFA supplementation may attenuate oxidative stress parameters after exercise.

## Methods

### Subjects

Participants (adult male and female subjects with T2DM) were recruited via advertising on local newspapers and workplaces. The exclusion criteria were: in use of aspirin or insulin, HbA1c above 9.0%, dyslipidemia or having a diagnosis of any complication of diabetes mellitus. The study protocol was in accordance with the Helsinki declaration and was approved by the Clinics Hospital of Porto Alegre (HCPA) Ethics and Research Committee (06–222). All subjects gave written, informed consent before participation.

#### Study design

**Fig. 1:**
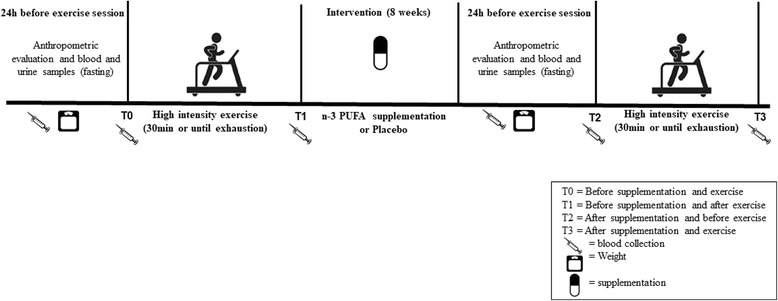
Flowchart of experimental sessions

This study has a randomized, double blind, parallel group design. Initially, physical evaluations were carried out, including interview with an expert physician and aerobic power test with electrocardiogram. After that, participants attended the outpatient clinic on four occasions: 1) Anthropometric evaluation and blood and urine collections in fasting; 2) First sub-maximal exercise test and beginning of supplementation; 3) Blood and urine sampling (fasting) after 8 weeks of supplementation, and 4) Second sub-maximal exercise test. Figure 1 illustrates the flowchart of experimental sessions.

### Anthropometric evaluation

During their first visit, the following anthropometric parameters were evaluated: body mass, height, circumferences and skinfolds. Body mass (kg) and height (m) was determined by a digital scale coupled to a stadiometer Welmy® (W 110 H, Santa Bárbara d’Oeste, SP, Brasil), with accuracy of 0.1 kg and 0.01 cm, respectively. Body mass index (BMI, kg/m^2^) and waist-to-hip ratio was calculated according to World Health Organization [17]. Body fat percent was determined by the Jackson & Pollock formula [18] and later converted to fat percentage, according to formula proposed by Siri [19]. All measurements of skinfolds were made on the right side of the body by using a compass (Cescorf®) with accuracy of 0.1 mm. After the supplementation period, body weight was evaluated.

### Blood and urine collection

Blood and urine were collected before and after supplementation period, after 12 h fasting, for analysis of total cholesterol and fractions, triglycerides, HbA1c and glycemia and albuminury (urine), 24 h before each exercise session. In the day of submaximal exercise, subjects were in the fed state, and blood collect occurred immediately before and after exercise (See Fig. 1).

### Exercise protocols

Peak oxygen consumption (VO_2_) and carbon dioxide production (VCO_2_) were measured breath by breath during the exercise via an automated ergospirometer metabolic cart (CPX-D, Medical Graphics Corporation, USA). The equipment was calibrated before each exercise test according to the manufacturer’s instructions. O_2_ and CO_2_ analyzers were calibrated using gas standards of known concentrations before each exercise test.

The maximal exercise tests were carried out on a cycle ergometer (The Byke, Cybex USA). All tests were started at a 25 W load, with 25 W/min increments until exhaustion, followed by a 3-min recovery with a 25 W load. Cycling should be kept between 60 to 90 cpm, on the subject’s most comfortable cadence [20]. Volunteers were verbally encouraged to achieve their best performance during the maximal exercise test. The criteria used to verify maximal effort included a respiratory gas exchange ratio ≥ 1.15, a final peak heart rate at ≥95% of the age-predicted maximum (220-age) and/or a VO_2_ plateau through systematic increases in the workload [21]. Anaerobic threshold was determined by the second ventilatory threshold (VT2) [22].

All high intensity sub-maximal tests were performed in the morning, 2 h after the subject’s usual breakfast. Volunteers were asked not to have high-intensity physical activity, caffeine consumption or alcohol ingestion 24 h before the tests. The tests were performed at an intensity of VT2, monitored by VO_2_ during exercise. After a 3-min rest, the test started. During the first 5 min, the load was increased to approximately 55% of VT2. The load was then adjusted to reach 100% of VT2. The subjects continued the exercise for 30 min or until exhaustion [23].

### Supplementation

Supplementation was given to the subjects in pharmacy manufactured capsules. The placebo capsules contained 500 mg of gelatin. The intervention group received n-3 PUFA-enriched capsules containing 180 mg of eicosapentaenoic acid (EPA), 120 mg of docosahexaenoic acid (DHA) and 2 mg of vitamin E (alpha-tocopherol) [Naturalis®, Brazil]. A low concentration of vitamin E was used in the formulation to prevent n-3 PUFA oxidation, as previously recommended [24]. Subjects in both groups were asked to consume three capsules a day (at breakfast, lunch and dinner), for 8 weeks.

### Biochemical analyses

Venous blood samples were taken after fasting from the antecubital vein in EDTA coated (for plasma) or plain (for serum) tubes using standard aseptic techniques. Urine samples for measurement of albuminuria also were collected only before and at the end of the supplementation period. Total cholesterol, HDL-cholesterol, triglycerides and glucose were measured by enzymatic colorimetric assays; Friedewald’s formula was used to calculate for LDL-cholesterol fraction [25]. Glycated hemoglobin (HbA_1C_) was measured by an immunoassay; urinary albumin was measured by immunoturbidimetry.

Before and after each submaximal test, blood samples were immediately centrifuged at 4 °C, 4000 rpm, for 5 min, and plasma or serum were separated and stored at − 75 °C for further analysis. We analyzed hs-CRP after each testing using nephelometry. Lipid Peroxidation was assessed by two distinct techniques: 1) Plasma F2-isoprostanes analysis, using a commercial kit (Cayman®), and 2) TBARS analysis, based in the thiobarbituric acid reactivity with malondialdehyde, a lipid oxidation end-product [26]. Non-enzymatic plasma antioxidant properties were assessed by total reactive antioxidant potential (TRAP), determined by chemiluminescence [27]. Superoxide dismutase (SOD) activity was measured indirectly through the inhibition of adrenaline auto-oxidation to adrenochrome, as described by Bannister [28]. Plasma protein was determined as described by Lowry et al. [29]. Uric acid was determined by a commercial kit Uricostat enzymatic AA, Wiener Lab®, Rosario, Agentina).

### Statistical analysis

Data were analyzed on SPSS 25.0 for Windows (IBM, USA). Normality was assessed by the Shapiro-Wilk test, and Levene’s test was used for homoscedasticity. For baseline value comparison between groups, Student’s *t-*test for independent samples or the Mann-Whitney test were performed. A generalized estimating equation (GEE) followed by Bonferroni’s post hoc test was used to verify supplementation and exercise effects adjusted for age, gender, duration of diabetes and use of drugs. The GEE model for each outcome was based on the goodness of fit. The normality of the residuals was verified by normal Q-Q plot. Some cardiometabolic variables were transformed due to the high variability among individuals, using a) logarithmic transformation for hs-CRP and F2-isoprostanes, b) square root transformation for albuminuria, uric acid and SOD, and c) inverse transformation for triglycerides and HbA_1C_. Cohen’s d was used to verify the effect size of the means. The accepted significance level was *p* < 0.05. Data were reported as mean ± standard deviation, and difference mean and 95% confidence interval (95% CI).

## Results

**Fig. 2:**
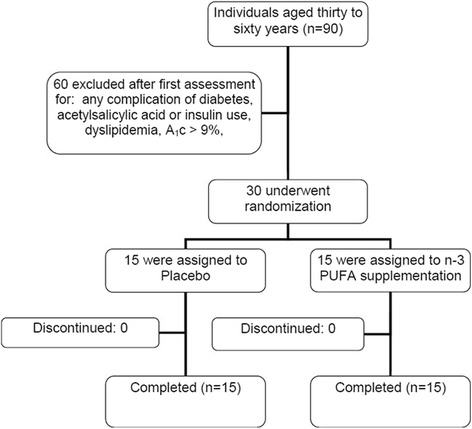
Flow diagram of patient recruitment and randomization

Figure 2 shows the flow diagram of patient recruitment and randomization. Of the 90 individuals who manifested interest in participating in the study, 60 were excluded based on the adopted criteria. In total, 30 individuals performed all baseline assessments, and all subjects completed the intervention. The characteristics of the participants are presented in Table 1. There were no differences in age, diabetes duration, weight, height, body mass index, percentage of body fat and peak oxygen consumption between the groups. Waist to hip ratio was higher in male subjects in the n3-PUFA group (*p* = 0.03).

**Table 1:** Baseline characteristics of the study groups

	n-3 PUFA (*n* = 15)	Placebo (*n* = 15)
N	15	15
Sex (male/female)	8/7	4/11
Age (years)	50.47 ± 6.06	50.67 ± 6.70
Diabetes Duration (years)	6 (2–15)	8 (1–25)
Drug Treatment (*n*)		
Diet only	3	3
Oral hypoglicemic agents		
Metformin	8	4
Sulfonylureas	3	0
Combined	1	8
Antihypertensive (*n*)		
Yes/No	5/10	8/7
Weight (kg)	77.27 ± 11.65	72.49 ± 7.66
Height (m)	1.65 ± 0.08	1.59 ± 0.09
Body Mass Index (kg.m^− 2^)	28.22 ± 2.95	28.81 ± 4.10
Body fat (%)	32.93 ± 8.88	33.79 ± 10.07
Waist-to-hip ratio		
Men	0.97 ± 0.05^a^	0.88 ± 0.05
Women	0.83 ± 0.08	0.87 ± 0.05
VO_2peak_ (ml/kg/.min)	22.87 ± 5.42	22.10 ± 6.87
Maximal heart rate (bpm)	151.13 ± 20.29	153.87 ± 19.98

^a^*p* = 0.03 with Student’s *t-*test

After the supplementation period, individuals did not change your body weight (0.94 ± 0.36% in n-3 PUFA supplementation and 0.75 ± 0.25% in placebo group, respectively, *p* > 0.05 for both). Table 2 shows the biochemical parameters before and after supplementation period. n-3 PUFA supplementation significantly reduced triglycerides by 17% (*P* = 0.005), compared with baseline values, and by 26% when compared with the placebo group (*P* = 0.05). No other interaction condition vs time was observed in the experimental groups.

**Table 2:** Cardiometabolic parameters in response to n-3 PUFA or placebo supplementation, in baseline and after 8 weeks (follow up)

	n-3 PUFA (*n* = 15)			Placebo (*n* = 15)			
	Baseline	Follow-up	Diff. Mean (95% CI)	Baseline	Follow-up	Diff. Mean (95% CI)	*P* value*
HbA1c (%)	6.11 ± 0.57	6.47 ± 0.86		7.01 ± 1.06	7.40 ± 1.72		
Log HbA1c (%)^b^	0.17 ± 0.01	0.16 ± 0.02	−0.008 (− 0.014 to − 0.002)	0.14 ± 0.02	0.14 ± 0.02	− 0.005 (− 0.020 to 0.010)	0.752
Glucose (mg/dl)	134.7 ± 10.1	131.1 ± 6.7	− 3.5 (− 0.01 to 4.7)	130.8 ± 5.5	122.8 ± 4.6	−8.0 (− 2.5 to 9.2)	0.382
Triglycerides (mg/dl)	136.5 ± 75.9	113.3 ± 71.4		152.9 ± 59.5	153 ± 56.4		
Log Triglycerides (mg/dl)^b^	0.009 ± 0.004	0.011 ± 0.005	0.002 (0.000 to 0.004)^**^	0.008 ± 0.003	0.008 ± 0.003	0.000 (− 0.001 to 0.001)	**0.005**
Total Cholesterol (mg/dl)	182.4 ± 41.3	177.1 ± 44.7	− 5.8 (− 17.8 to 6.1)	192.3 ± 38.2	193.3 ± 40.4	0.7 (− 15.8 to 17.2)	0.385
HDL-cholesterol (mg/dl)	49.7 ± 15.9	52 ± 15	2.3 (− 0.5 to 5.1)	51.7 ± 12.2	53.9 ± 12.7	2.1 (− 3.0 to 7.2)	0.904
LDL-cholesterol (mg/dl)	105.4 ± 35.3	102.4 ± 41.4	− 3.9 (− 16.4 to 8.6)	110.0 ± 35.6	108.8 ± 41.7	−1.4 (− 16.9 to 14.1)	0.732
Albuminuria (mg/dl)	9.67 ± 6.06	8.89 ± 7.04		5.64 ± 3.52	3.99 ± 2.25		
Log Albuminuria (mg/dl)^a^	2.25 ± 0.59	1.92 ± 0.49	− 0.33 (− 0.73 to 0.07)	2.91 ± 0.82	2.74 ± 1.04	− 0.16 (− 0.93 to 0.60)	0.426

Values are presented as mean ± standard deviation. *Models were adjusted for age, gender, duration of diabetes and use of drugs, Logarithmic transformation, ^a^Square root transformation, ^b^Inverse transformation, ^**^(*p* = 0.004 compared to before supplementation

*p* value in bold was statiscally significant

Regarding submaximal exercise tests, there were no differences in time duration in both groups before (14 ± 5.02 min and 15 ± 6.1 min) and after supplementation (15 ± 4.7 min and 13 ± 5.8 min) for n-3 PUFA supplementation and placebo groups, respectively (*p* > 0.05). Table 3 shows the results about oxidative stress and inflammation parameters after supplementation and submaximal exercises. No significant changes were observed for TBARS nor F2-Isoprostanes concentrations (primary outcome). In the n-3 PUFA supplementation group, a statistical significant interaction (condition vs time) was observed for TRAP count values, with decrease after supplementation and exercise. Cohen’s *d* effect size was consider high for this mean difference (d ≥ 0.8). Although no statistically significant interaction was found for SOD levels (*p* = 0.095), the n-3 PUFA supplementation group presented an increase after supplementation (*p* = 0.002, Cohen’s *d* effect size = 0.49), with a tendency in their concentrations after exercise. There was no significant difference for hs-CRP between the groups.

**Table 3:** Variables of oxidative stress between the groups (n-3 and placebo) and times (pre-exercise and post-exercise)

	n-3 PUFA (*n* = 15)				Placebo (*n* = 15)				
	Baseline		Follow-up		Baseline		Follow-up		
	Pre-exercise	Post-exercise	Pre-exercise	Post-exercise	Pre-exercise	Post-exercise	Pre-exercise	Post-exercise	*P* value*
us-CRP (mg/dL)	2.88 ± 3.2	3.10 ± 3.35	2.37 ± 2.42	2.56 ± 2.66	3.36 ± 3.39	3.63 ± 3.61	3.19 ± 3.26	3.43 ± 3.51	
Log us-PCR (mg/dL)^a^	0.50 ± 0.29	0.53 ± 0.3	0.45 ± 0.28	0.47 ± 0.28	0.51 ± 0.28	0.49 ± 0.27	0.53 ± 0.29	0.50 ± 0.29	0.712
Diff. Mean (95% CI)	0.02 (0.01 to 0.03)		0.02 (0.01 to 0.04)		0.03 (0.01 to 0.04)		0.02 (0.01 to 0.03)		
Cohen’s *d*	0.10		0.07		0.07		0.10		
TBARS (pmolTBARS/mgPTN)	40.2 ± 20.9	36.7 ± 17	29.7 ± 9.9	32.5 ± 11.8	38.4 ± 19.2	40.0 ± 20.5	37.5 ± 17.7	33.1 ± 11.8	0.052
Diff. Mean (95% CI)	−3.8 (− 10 to 2.4)		− 2.9 (−1.6 to 7.4)		1.62 (− 1.03 to 4.28)		−4.4 (− 10.7 to 1.8)		
Cohen’s *d*	0.18		0.26		0.08		0.28		
F2-isoprostanes (ng/mL)	325.9 ± 314.5	262.2 ± 113	267 ± 124.9	255.1 ± 134.1	284.5 ± 190.1	319.9 ± 149.1	282.1 ± 147.3	226.1 ± 114.4	
Log F2-isoprostanes (ng/mL)^a^	2.4 ± 0.39	2.36 ± 0.26	2.34 ± 0.34	2.32 ± 0.32	2.37 ± 0.29	2.45 ± 0.25	2.37 ± 0.28	2.30 ± 0.2	0.438
Diff. Mean (95% CI)	−0.05 (− 0.19 to 0.10)		−0.02 (− 0.19 to 0.16)		0.08 (− 0.05 to 0.21)		−0.07 (− 0.18 to 0.04)		
Cohen’s *d*	0.12		0.06		0.26		0.28		
Uric Acid (mg/L)	47.5 ± 16.0	48.6 ± 17.7	47.9 ± 19.1	46.9 ± 17.2	38.6 ± 8.1	39.8 ± 7.0	36.2 ± 6.9	36.6 ± 7.3	
Log Uric Acid (mg/L)^b^	6.71 ± 0.91	6.77 ± 1.05	6.70 ± 1.12	6.64 ± 0.99	6.24 ± 0.71	6.36 ± 0.74	6.04 ± 0.64	6.09 ± 0.74	0.639
Diff. Mean (95% CI)	0.06 (− 0.14 to 0.26)		−0.06 (− 0.19 to 0.08)		0.12 (− 0.13 to 0.38)		0.04 (− 0.13 to 0.22)		
Cohen’s *d*	0.06		0.06		0.17		0.07		
TRAP (contains/min)	148,779 ± 51,653	158,420 ± 51,275	182,404 ± 29,313	148,520 ± 31,339	168,614 ± 36,302	166,249 ± 44,925	152,720 ± 347,681	155,678 ± 42,763	**0.023**
Diff. Mean (95% CI)	9641 (− 20,068 to 39,351)		−33,884 (− 56,976 to − 10,793)^**^		− 2366 (− 29,426 to 24,695)		2957 (−20,724 to 26,639)		
Cohen’s *d*	0.19		1.12		0.06		0.01		
SOD (USOD/mgPTN)	1.90 ± 0.99	2.44 ± 1.34	4.04 ± 2.33	4.60 ± 2.62	3.08 ± 1.74	3.06 ± 1.34	3.30 ± 1.23	3.90 ± 1.75	
Log SOD (USOD/mgPTN)^b^	1.34 ± 0.36	1.52 ± 0.38	1.96 ± 0.60	2.06 ± 0.57	1.68 ± 0.37	1.70 ± 0.29	1.77 ± 0.32	1.90 ± 0.42	0.095
Diff. Mean (95% CI)	0.18 (− 0.05 to 0.41)		0.11 (− 0.46 to 0.67)		0.02 (− 0.23 to 0.26)		0.13 (−0.20 to 0.45)		
Cohen’s *d*	0.49		0.17		0.06		0.34		

Values are presented as mean ± standard deviation. * Models were adjusted for age, gender, duration of diabetes and use of drugs, ^a^ Logarithmic transformation, ^b^ Square root transformation, ^**^ *p* = 0.004 compared to before supplementation

*Abbreviations: hs-CRP* high sensitivity C-reactive protein, *TBARS* thiobarbituric acid reactive substances, *SOD* superoxide dismutase

*p* value in bold was statiscally significant

## Discussion

The main finding of this study was that 8 weeks of n-3 PUFA supplementation reduces TRAP concentration after a high intensity exercise, with no other statistically significant changes in other oxidative stress parameters or inflammation. Additionally, as previously demonstrated, n-3 PUFA supplementation reduced triglycerides levels. Our hypothesis was partially rejected, due to the fact that our primary outcomes (TBARS and F2-isoprostanes) were not attenuated by n-3 PUFA supplementation.

As previously mentioned, physical exercise is considered a powerful tool to prevent and to treat diabetes and the associated comorbidities. Nowadays, due to the lack of time, many people are using high intensity exercise sessions (with a lower volume), as an alternative, to improve their health, metabolic function and body composition. However, high intensity exercise can induce a transient inflammatory state and increase oxidative stress [10, 11] that may promote, in diabetic population, an undesired effect. The increase in VO_2_ (load dependent) caused by exercise can lead to increased blood and tissue levels of reactive oxygen species [30]. Laaksonen et al. [31] and Atalay et al. [32] showed increased reactive oxygen species production after 40 min of sub-maximal exercise (intensity equivalent to 60% of VO_2peak_) in type 1 diabetic subjects. In our study, exercise performed with intensity equivalent to 70–80% of VO_2peak_ was not able to increase the production of lipoperoxides as measured by TBARS and F2-Isoprostanes (primary outcomes). If exercise intensity was to be an issue, our data are corroborated by the findings of Davison et al. [33], that did not observe differences in the concentrations of malondialdehyde even after strenuous exercise. In addition, the lack of changes in TBARS and F2-isoprostanes may be attributed, at least in part, by the shorter period of supplementation of the present work in comparison with others.

We hypothesized that including n-3 PUFA to our subjects would induce benefits when they exercise at high intensity, however, with except of changes in TRAP, our intervention fail to change levels of lipoperoxidation, SOD and UA. Accinni and colleagues [34] also found an increase in TRAP in dyslipidemic volunteers supplemented with n-3 PUFA for 4 months, however, with a significant reduction in the levels of TBARS (*P* = 0,002). After treating healthy volunteers with 3.6 g/day of n-3 PUFA, Nalsen et al. [16] did not observe changes in the values of TRAP; however, they found a significant (*P* = 0.015) reduction in lipoperoxidation as measured by F2-isoprostanes concentration. The difference in results among studies may be related to different exercise protocols, duration of the tests, and different methods employed when measuring the oxidative stress markers.

Regarding SOD and UA, our secondary outcomes, there were no significant changes after the exercise, similarly to the results described by Laaksonen et al. [31] and Atalay et al. [32]. If our subjects were more obese or dyslipidemic, the results could be different (49). There is still very limited information on the effects of exercise in acute oxidative stress in subjects with type 2 diabetes. The trend for increased activity of SOD after supplementation with n-3 PUFA was not reported in the work of Kesavulu et al. [4]. However, Smaoui et al. [35] showed a positive correlation between SOD activity and concentrations of n-3 PUFA in erythrocytes. Erdogan et al. [36] also found increased activity of SOD in rats after 0.4 / kg body weight fish oil supplementation for 30 days. This increase in SOD and TRAP could be explained by the formation of cyclopentenone prostaglandins. Supplementation with n-3 PUFA, particularly with EPA and DHA, has been documented to trigger a momentary lipoperoxidation process, thus leading to the formation of prostaglandins, particularly cyclopentenone prostaglandins. These are responsible for the dissociation of Keap1/Nrf2 (NF-E2-related factor). Nrf2 is the main transcription factor for the activation of the expression of antioxidant enzymes (e.g. SOD, catalase, glutathione reductase) as well as phase 2 enzymes (e.g. glutathione S-transferase, NADPH oxidase) and thus also increases TRAP [37]. The production of lipoperoxide is not considered enough to cause damage to the body, but important to stimulate the defense systems.

Subjects who develop T2DM have high levels of inflammatory markers such as hs-CRP when compared to healthy subjects [37, 38]. Supplementation with n-3 PUFA has been suggested to minimize the inflammatory processes [39]. However, our study failed to detect any significant change in the plasma levels of hs-CRP after supplementation with n-3 PUFA. Our findings corroborate the data published by Madsen et al. [40] who worked with healthy subjects supplemented with 2 or 6.6 g / day of n-3 PUFA, for a period of 12 weeks. Both groups showed no change in the values of hs-CRP. Mori et al. [15] and Fatemeh Azizi-Soleiman et al. [41] also found no change in plasma levels of CRP after supplementation with EPA or DHA, for 6 weeks and 8 weeks, respectively, in type 2 diabetic subjects. Other studies also have shown that either EPA or DHA reduce in vivo oxidant stress without changing markers of inflammation, in treated, hypertensive, type 2 diabetic subjects [15]. Finally, despite the lack of changes of hs-CRP, we cannot exclude the potential anti-inflammatory effects of our supplementation, since we did not look at other inflammatory markers such as TNF-α, IL-8, eHSP70 etc. This is a limitation of our study.

Plasma glucose and HbA1c did not change significantly after the period of supplementation. Some studies reported increased levels of glucose, HbA1c and low insulin activity, in type 2 diabetes, after the consumption of large amounts of fish oil (around 10 g/day) [42]. However, when of n-3 PUFA is given in smaller quantities (1 to 4 g/day), no changes in glycemic parameters are observed [43]. In our study, a significant reduction (17%) in the values of plasma triglycerides was observed after the period of supplementation with n-3 PUFA. These results can be explained by the reduction of fractions of VLDL - thus minimizing the hepatic production of triglycerides - and a possible reduction of free fatty acids [44]. As in other studies, there were no significant changes in other lipid parameters [43, 45–47]. This can be partially explained by the fact that the diabetic subjects in the present study were not dyslipidemic, and were relatively lean (overweight on average). This differs from many studies in the literature. In a meta-analysis covering studies between 1966 and 2006, Hartweg et al. [48], showed that n-3 PUFA supplementation is, indeed, efficient to reduce triglycerides and VLDL levels, without changing other lipid sub fractions. In contrast, Abe et al. [49], after 6 weeks of supplementation with 4 g/day n-3 PUFA, found an 11% reduction in total cholesterol and a 14% increase in HDL. These changes are not a consensus in the available literature. In addition, a 4-h infusion of intralipid (n-3 FA) failed to affect insulin sensitivity, insulin secretion, or markers of oxidative stress in subjects with T2DM [50]. It could explain the lack of changes in the glycemic profile.

To the best of our knowledge, this is the first study to examine the interactions between n-3 PUFA supplementation and high-intensity exercise on oxidative stress and inflammation in subjects with T2DM. However, we recognize that the study has several limitations. First, the sample size was small, although the effect size on the variables with statistical significance was high. So, at this point, should be interpreted with caution before being extrapolated to the general population. Second, we have only studied acute effect of high intensity exercise; therefore, it is not possible to state that the effects would be similar after other intensities of exercise. Finally, we did not analyze the supplement offered to the participants, so we cannot be 100% sure if the product provides the amount of n-3 PUFA. We believe in the suitability of the company, but we suggest that future studies test the product before the beginning of the trial. Despite some limitations, the strength of this study is that we analyzed a large number of variables related to oxidative stress, including two measurements of lipoperoxidation (TBARS and F2-siprostanes) and measurements of enzymatic (SOD) and non-enzymatic antioxidants (uric acid and TRAP). Thus, this study contributes to narrow the gap in the literature on the effects of antioxidants nutrients in oxidative stress parameters after acute exercise. Further clarification is needed regarding the clinical relevance of the n-3 PUFA supplementation for T2DM in different intensities of exercise.

## Conclusions

Eight weeks of supplementation with n-3 PUFA did not attenuate oxidative stress parameters after a high intensity exercise in T2DM subjects, with exception to TRA*P* values. On the other hand, n-3 PUFA cause reduction of triglyceride levels, at rest, without altering membrane oxidative damage or inflammation (as assessed by hs-CRP). Studies including other inflammation markers such as IL-8, MCP-1, eHSP70, or others, are required to better assess this response. Likewise, longer periods of observation and/or and other intensities of exercise may be necessary to induce significant increments in oxidative stress parameters in diabetic patients.

## Abbreviations

DHA: Docosaexaenoic acid
EPA: Eicosapentaenoic acid
HR: Heart rate
hs-CRP: High sensitivity C-reactive protein
n-3PUFA: Polyunsaturated fatty acids omega-3
SOD: Superoxide dismutase
T2DM: Type 2 diabetes mellitus
TBARS: Thiobarbituric acid reactive substances
TRAP: Total reactive antioxidant potential
VCO_2_: Carbon dioxide production
VO_2_: Oxygen consumption
VT2: Second ventilator threshold
